# Sleep disorders in correctional officers: cross-sectional study

**DOI:** 10.5935/1984-0063.20210027

**Published:** 2022

**Authors:** Glécia Lemos Bezerra, Fernando Martins Carvalho, Rita de Cássia Pereira Fernandes, Kionna Oliveira Bernardes Santos

**Affiliations:** Federal University of Bahia, Postgraduate Program in Health, Enviroment, and Work - Salvador - Bahia - Brazil.

**Keywords:** Sleep, Prisons, Working Conditions, Violence, Mental Health

## Abstract

**Introduction:**

Within the prison environment, where strict surveillance and prompt decision-taking are essential to maintain security, poor sleep may be hazardous to correctional officers. This study aims to estimate the prevalence of and identify factors associated with severe sleep disorders in correctional officers.

**Material and Methods:**

An exploratory cross-sectional study comprised all correctional officers in a prison complex in Salvador city, Brazil. Information about sleep quality, sociodemographic and occupational aspects, lifestyle habits, and common mental disorders (self-reporting questionnaire-20 score ≥7 points) were collected via a self-administered questionnaire. Severe sleep disorder was defined as a score ≥31 points according to the mini sleep questionnaire. The measure of association used was the prevalence ratio (PR). Prevalence ratios were estimated by using a Cox multivariate regression model. The final adjusted model only included the variables that presented a prevalence ratio ≥1.20.

**Results:**

The prevalence of severe sleep disorders in the 374 correctional officers was 55.3%, and was strongly (prevalence ratio ≥1.20) associated with number of stressful activities at work (RPs=1.24, 1.19, and 1.17), number of attack and threat events against the correctional officers over the last 12 months (RPs showing gradient, 1.11, 1.24, and 1.41), common mental disorders (RP=2,24), and non-White skin color (RP=1,37).

**Conclusion:**

This study found high prevalence of severe sleep disorders in correctional officers, associated with impairment of their mental health, skin color, and, particularly, with situations of stress and violence at work. These factors must be taken into account when planning and providing health care to these workers.

## INTRODUCTION

In the prison system context, correctional officers are exposed to several sources of stress, including a closed coercitive work enviroment, the need to deal with violence and perform ardous tasks, constant vigilance about order and security, difficult work schedules, and obedience to hierarquical relationships^1^. A review about the psychological distress and work stress of correctional officers identified 40 articles, with 22 focused on stress, 12 on burnout, and eight on psychic suffering. Sixteen publications came from the United States of America; four studies came from Latin America, all of them from Brazil^2^. In July 30, 2017, the Brazilian penitentiary system had 1,507 prisons, 706,619 prisoners, and 80,350 correctional officers^3^. The functioning of this system is hampered by inadequate public policies, corruption, shortage of material and human resources, overcrowding, and violence^4^.

Correctional officers present a high prevalence of diabetes, cardiovascular diseases, stroke, arterial hypertension, metabolic disorders, obesity, increased risk of physical injuries, burnout, depression, anxiety, suicide, infectious diseases, and sleep disorders^5^.

Insufficient sleep can affect many aspects of individual health, causing daytime sleepiness; emotional problems; cognitive impairment; effects on body weight; changes in circadian rhythm, glucose metabolism, and inflammatory response; and negative impacts on the cardiovascular, reproductive, and immunological systems. In the community context, sleep deprivation increases the probability of occupational and traffic accidents, decreases sociability, and performance and productivity at work^6^. In occupational environments, sleep deficiency affects worker productivity, and health and safety, and leads to high economic burdens. Problems related to insufficient sleep hours have been related to the loss of estimated 717 billion dollars in 2020 in five countries (United States of America, Canada, United Kingdom, Germany, and Japan)^7^.

In the prison environment, where strict surveillance and prompt decision-taking are essential for maintaining security, sleep deprivation and consequent fatigue can threaten correctional officers’ life, since it impairs their ability to evaluate risks^8^. The prison environment is dangerous and interactional. Correctional officers must be able to defuse tension and maintain calm among prisoners, requiring to only use necessary amounts of force to maintain safety without abusing prisoners. Correctional officers sleep disorders can lead to mistakes, irritation, and excessive responses to problems, resulting in abuse or unnecessary harm to prisoners.

Correctional officers are frequently affected by sleep disorders. A study with 355 prison workers from the State of Washington, USA, reported high prevalence of (27.6%), and insomnia (44.8%), according to the Pittsburg sleep quality index^5^. A nationwide, representative study among correctional officers in France reported a 41.8% prevalence of sleep disorders, evaluated by a specific questionnaire^1^.

The objective of this study is to estimate the prevalence of and identify factors associated with severe sleep disorders among correctional officers in a Brazilian prison complex.

## MATERIAL AND METHODS

A cross sectional exploratory study including all 571 correctional officers assigned to the public State Penitentiary Complex in the city of Salvador, Brazil. All correctional officers studied were employees of the State Justice Department, selected by an open competition exam that includes physical and psychological evaluation of their aptitute for the profession. These employees have full labor rights assured, including sick leave.

The correctional officers were approached during working time and participated voluntarily in the study. After explaining the objectives, participants signed an informed consent form. All information relating to the participants was confidential. The study was approved by the ethics committee in research on human beings of the Medical School of Bahia for opinion number 2.464.066 and amendment 2.824.557, and carried out in accordance with the Helsinki declaration.

Data collection occurred between September and December 2018. Correctional officers were excluded from the study if were on sick leave (17); on a ninety-day sabbatical (15); who retired during data collection (6); where there was a lack of information about the unit they were assigned to (30); who had been substituted by another correctional officer during a whole month (37); who had been transferred to another work station or were working for the union (15); or who deviated from their prescribed work position (4), resulting in 447 eligible individuals. Of these, 46 were excluded because they refused to participate in the study after three consecutive invitations, and 27 because they provided incomplete answers to the study questionnaire. In the end, the study population comprised 374 individuals, corresponding to 83.7% of all eligible officers.

A self-administered questionnaire containing sociodemographic and occupational information, lifestyle habits, common mental disorders, and sleep quality was handed over to the correctional officer that took 20-30 minutes to complete it.

Subjective sleep quality was assessed by the mini sleep questionnaire (MSQ), translated into Brazilian Portuguese^9^. The Brazilian MSQ contains ten questions on sleep-related aspects, such as difficulty falling asleep, mid-sleep awakening, use of hypnotic medication, excessive daytime sleepiness, feeling tired on waking up in the morning, snoring, waking up too early, headaches on awakening, falling asleep during the day, and excessive movements during sleep. The answers are provided in Likert-type scales varying from 1 (never) to 7 (always). The total sum of scores provides an estimate of sleep quality, classified as: good sleep quality (10-24 points); mild sleep disorders (25-27 points); moderate sleep disorders (28-30 points); and severe sleep disorders (≥31 points)^10^. The study outcome variable (severe sleep disorders) was dichotomized using a cutoff of ≥31 points.

A study with 1,830 undergraduate students assessed the psychometric properties and reliability of the MSQ Italian version, and recommended it as a useful screening tool for insomnia and hypersomnia. Cronbach’s alpha was 0.77^11^. A study of Brazilian undergraduate students reported the MSQ to be a good tool for assessing sleep disorders, with adequate reliability (Cronbach’s alpha of 0.770) and test-retest reliablity^10^.

The following occupational characteristics were investigated: time worked as correctional officer, prison unitiy (Feminine Unit; Specialized Unities - the entrance gate to the prison complex, the prisoners healthcare center, and the special operations group); Closed Prison Unit, for prisoners in the closed prison regime; and the Pretrial Unit, for custody of pretrial inmates), working position, contact with prisoners, training for the current post, shift work, having another job, membership of a union or other professional association, witnessed prisoner escape, carries firearm on duty, has discharged firearm on duty, has been threatened by crime factions.

The correctional officers were asked about nine stressful activities at their work: prisoner admission; opening prison gate; closing prison gate; visiting hours; cell search; prisoner search; prisoner counting; escorting prisoner during indoor activities; and escorting prisoner during outdoor activities. These questions were answered as 0 = No or 1 = Yes. A new ordinal variable was built, by grouping the number of stressful activities as; 0-1; 2-4; 5-7; and 8-9.

The attack or threat events against the correctional officer over the last 12 months were evaluated by three questions addressing violence perpetrated by prisoner; by relative, companion, or neighbor; or by the correctional officer’s chief or colleague, answered on a no/yes basis. A new variable was built to represent the total number of attack or threat events, coded as 0, 1, 2, and 3. Information was collected about age (23-59/60-72 years), sex (male/female), skin color (White/Non-White) civil status (married or stable partnership/other), having children (yes/no), schooling (higher education or graduatio/high school), weekly practice of physical activity (one or more days per week/none), regular leisure activities (yes/no), smoking (yes, for current smokers/no, for non- and ex-smokers). Alcoholism was detected by using CAGE questionnaire, applying a ≥2 positive answers cutoff point^12^. Common mental disorders were evaluated by the *self-reporting questionnaire* (SRQ-20), applying a ≥5 cutoff point for men and ≥7 for women^13^.

### Statistical analysis

In bivariate analysis, prevalence ratio (PR) was calculated for severe sleep disorders according to the strata of independent variables. The variables for entry in the Cox regression model were then selected on the basis the magnitude of the association (PR >1.20), and according to theoretical plausibility and the epidemiological evidence in the literature. This evidence led to the inclusion of the shift work variable in the multivariable models. Shift work is associated with a high prevalence of sleep disorders^14,15^. Variables were inserted in the Cox model by using the ENTER method. Following the initial selection, the four remaining independent variables as well as shift work variable were again included in the final Cox regression model, in order to calculate their respective prevalence ratios. Although arbitrarily defined as a benchmark, the adjusted PR>1.20 evaluates effect size, instead of statistical significance. According to Rothman (2014)^16^, epidemiological studies should focus on the effect size (the magnitude of the associations), instead of statistical testing.

Cox regression is generally used to analyze longitudinal studies in which the response is the number of episodes of an event over a given time. However, Cox regression is an adequate technique to analyze cross-sectional studies^17^. In this case, the follow-up time should be set as 1 for all participants, as a strategy to obtain PR point estimates^18,19^. Further, Cox regression has the advantage to allow estimates of prevalence ratios instead of prevalence odds ratios^17^.

Since it was a census study, sampling procedures techniques were not used. Using statistical inference tests or calculating confidence intervals were therefore not appropriate^16,20,21^.

Data was processed and analyzed using SPSS (*Statistical Package of Social Science*) version 22.0.

## RESULTS

The prevalence of severe sleep disorders (MSQ≥31 points) among the 374 correctional officers was 55.3%. The prevalence of moderate sleep disorders, mild sleep disorders, and good sleep were 11.0%, 10.7%, and 23.0%, respectively.

Bivariate analysis revealed that prevalence of severe sleep disorders was strongly (PR>1.20) higher among correctional officers who belonged to the younger age group (23 to 59 years), declared their skin color to be non-White, did not practice physical activities, did not practice regular leisure activities, were smokers, present common mental disorders (Table 1), work in a prison unit that not the feminine, and have another job. Correctional officers who worked shifts had a 10% higher prevalence of severe sleep disorders than those who did not (Table 2). Three hundred and nineteen (85.3%) out of the 374 correctional officers worked shifts. The 24 x 72 hours shift was the most frequent work schedule (312/319 = 97.8%); four correctional officers worked in 12x24 hours shift, and three worked in 24x36 hours shift. Sleeping while on duty was not allowed.

**Table 1:** Severe sleep disorders according to sociodemographic, habits and common mental disorders characteristics of 374 correctional officers.

Characteristic	Severe sleep disorders				PR
	Yes		No		
	N	%	N	%	
Sex					
Feminine	37	59.7	25	40.3	1.09
Masculine	170	54.5	142	45.5	1
Age (years)					
23-59	180	57.1	137	42.9	1.20
60-72	27	45.6	30	54.4	1
Skin color					
Non-White	197	56.6	151	43.4	1.47
White	10	38.5	16	61.5	1
Schooling					
Higher education/graduation	133	56.4	103	43.6	1.05
High school	74	53.6	64	46.4	1
Civil status					
Married/Stable partnership	147	57.4	109	42.6	1.13
Others	60	50.8	58	49.2	1
Children					
Yes	152	56.3	118	43.7	1.06
No	55	52.9	49	47.1	1
Physical activity (days/week)					
None	102	65.4	54	34.6	1.36
One or more	105	48.2	113	51.8	1
Regular leisure activities					
No	54	78.3	15	21.7	1.56
Yes	153	50.2	152	49.8	1
Smoking					
Yes	17	65.4	9	34.6	1.20
Non-smoker/ex-smoker	190	54.6	158	45.4	1
Alcoholism					
Yes	26	61.9	16	38.1	1.13
No	181	54.5	151	45.5	1
Common mental disorders					
Yes	126	86.3	20	13.7	2.43
No	81	35.5	147	64.5	1

Notes: PR = Prevalence ratio.

**Table 2:** Severe sleep disorders according to occupational characteristics of 374 correctional officers.

Occupational characteristic	Severe sleep disorders				PR
	Yes		No		
	N	%	N	%	
Time worked as correctional officer (years)					
>10	105	55.3	85	44.7	1.00
0-10	102	55.4	82	44.6	1
Prison unit					
Closed prison	77	61.1	49	38.9	1.53
Specialized	26	55.3	21	44.7	1.38
Pretrial	86	50.3	85	49.7	1.26
Feminine	12	40.0	18	60.0	1
Working position					
Security	171	55.9	135	44.1	1.06
Coordenation/Administration/Diretory	36	52.9	32	47.1	1
Contact with prisoners					
Yes	168	55.8	133	44.2	1.04
No	39	53.4	34	46.6	1
Training for the current post					
No	66	51.6	62	48.4	0.90
Yes	141	57.3	105	42.7	1
Shift work					
Yes	179	56.1	140	43.9	1.10
No	28	50.9	27	49.1	1
Another job					
Yes	51	64.6	28	35.4	1.22
No	156	52.9	139	47.1	1
Union or association membership					
Yes	74	59.2	51	40.8	1.11
No	133	53.4	116	46.6	1

**Note:** PR = Prevalence ratio.

The prevalence of severe sleep disorders was >1.20 and rose in proportion (1, 1.33, 1.36, 1.56, and 2.00) to the number of attacks or threats against the correctional officer over the last 12 months and among those who were threatened by criminal factions (Table 3).

**Table 3:** Prevalence ratio (PR) for severe sleep disorders according to stressful or violent situations at work of 374 correctional officers.

Stressful/violent situation	Severe sleep disorders				PR
	Yes		No		
	N	%	N	%	
Number of attacks or threats against correctional officer, last 12 months					
0	91	44.8	112	55.2	1
1	55	59.8	37	40.2	1.33
2	35	70.0	15	30.0	1.56
3	26	89.7	3	10.3	2.00
Carries firearm on duty					
Yes	43	56.6	33	43.4	1.03
No	164	55.0	134	45.0	1
Witnessed prisoner escape					
Yes	116	58.9	81	41.1	1.14
No	91	51.4	86	48.6	1
Has been threatened by crime factions					
Yes	76	66.7	38	33.3	1.32
No	131	50.4	129	49.6	1

**Notes:** PR = Prevalence ratio.

The prevalence of severe sleep disorders was strongly (PR>1.20) associated with the stressful acitivies visiting hours, cell search, prisoner counting, and escorting prisoners during indoors activities. The prevalence of severe sleep disorders was >1.20 and increased (1, 1.39, 1.48, and 1.67) according to the number of stressful activities at work (0-1, 2-4, 5-7, and 8-9, respectively) (Table 4).

**Table 4:** Prevalence ratio of severe sleep disorders according to stressful activities at work of 374 correctional officers.

Stressful activity at work	Severe sleep disorders				PR
	Yes		No		
	N	%	N	%	
Prisoner admission					
Yes	40	59.7	27	40.3	1.10
No	167	54.4	140	45.6	
Opening prison gate					
Yes	128	59.3	88	40.7	1.18
No	79	50.0	79	50.0	
Closing prison gate					
Yes	139	55.8	110	44.2	1.03
No	68	54.4	57	45.6	
Visiting hours					
Yes	58	65.9	30	34.1	1.27
No	149	52.1	137	47.9	
Cell search					
Yes	63	63.6	36	36.4	1.22
No	144	52.4	131	47.6	
Prisoner search					
Yes	64	57.7	47	42.3	1.10
No	143	54.4	120	45.6	
Prisoner counting					
Yes	108	63.9	61	36.1	1.32
No	99	48.3	106	51.7	
Escorting prisoner, indoors					
Yes	107	61.1	68	38.9	1.22
No	100	50.3	99	49.7	
Escorting prisoner, outdoors					
Yes	114	70.9	89	29.1	1.03
No	93	54.4	78	45.6	
Number of stressful activities at work					
0-1	28	59.4	41	40.6	1
2-4	103	43.7	80	56.3	1.39
5-7	53	39.8	35	60.2	1.48
8-9	23	32.4	11	67.6	1.67

**Notes:** PR = Prevalence ratio.

All 11 variables presenting PR>1.20 and the variable shift work were selected to compose a model analyzed by Cox regression. Four variables were still strongly (PR>1.20) associated with the dependent variable, after this first run. In the final model, the prevalence ratios adjusted by the multivariate model revealed that severe sleep disorders were strongly associated (PR≥1.20) with number of stressful activities at work (RPs=1.24, 1.19, and 1.17), number of attack and threat events against the correctional officers over the last 12 months (RPs showing gradient, 1.11, 1.24, and 1.41), common mental disorders (RP=2.24), and non-White skin color (RP =1.37). Shift work was not strongly associated with the outcome (RP=1.08) (Table 5).

**Table 5:** Prevalence ratios (PR) estimated by Cox regression model for factors associated with severe sleep disorders in 374 correctional officers.

Factor (referent)	Model with selected variables	Final model
	PR	PR
Age (60-72 years)	1.02	-
Physical activity (one or more days/week)	1.08	-
Regular leisure activities (Yes)	1.06	-
Smoking (No)	1.18	
Another job (No)	1.10	-
Has been threatened by crime factions (No)	1.10	-
Prison unit		
Feminine	1	-
Pretrial	0.85	-
Specialized	0.79	-
Closed prison	0.96	-
Number of stressful activities at work		
0 - 1	1	1
2 - 4	1.20	1.24
5 - 7	1.11	1.19
8 - 9	1.11	1.17
Number of attacks and threats against correctional officer over the last 12 months		
0	1	1
1	1.16	1.15
2	1.22	1.24
3	1.44	1.41
Non-White skin color (White)	1.34	1.37
Common mental disorders (No)	2.14	2.24
Shift work (No)	1.08	1.08

**Notes:** PR = Prevalence ratio.

The 27 individuals excluded from the study because did not provide valid answers to all questions of the study. These 27 correctional officers had lower prevalence of severe sleep disorders than the 374 individuals who had a complete set of information (33.3% versus 55.3%, respectively). These 27 individuals were similar to the 374 individuals effectively investigated in relation to the following characteristics: male (74.1% versus 83.4%, respectively), age group 23-59 years (88.9% versus 84.7%), non-White skin color (85.2% versus 93.0%), higher education/graduation schooling (66.7% versus 63.1%), and common mental disorders (27.5% versus 25.9).

## DISCUSSION

This study found a high prevalence of severe sleep disorders among correctional officers: 55.3%. This high prevalence justifies the use of prevalence ratio as the association measure and the Cox regression model in this study. The use of prevalence odds ratio obtained by the logistic regression model would overestimate the association measure^22^.

To the best of our knowledge, there are no comparable studies about sleep disorders among Brazilian correctional officers. However, studies have measured the frequency of sleep disorders in other Brazilian populations. In a random sample of the general population from the city of Presidente Prudente, Brazil, the prevalence of sleep disorders was 46.7% (95% CI: 43.1-50.2), using an MSQ≥25 cutoff point^23^. Among lecturers from Feira de Santana State University, Brazil, the prevalence of poor sleep quality was quite high, 61.3%, using MSQ≥28 cutoff point^24^, but, nevertheless, was lower than that found in our study, using the same ≥28 cutoff point: 66.3%. A population-based study in the city of Campinas, Brazil, assessed the sleep quality of with 1,998 people aged 20 or above, posing the single question: “How do you evaluate the quality of your sleep?”. The answers were expressed as: excellent/very good, good, regular, bad, or very bad. The combined percentage of “bad” and “very bad” responses was 29.1% (95% CI: 26.5-31.7)^25^. Studies using other questionnaires to measure sleep disorders found lower prevalences: 41.8% in France^1^ and 44.8% for insomnia and 27.6% for sleep apnea in the USA^5^.

The results of this study final model confirmed that the prevalence of severe sleep disorders was strongly associated with four factors: number of stressful activities at work, number of attack and threat events against the correctional officers over the last 12 months, common mental disorders, and non-White skin color.

This study found a positive gradient (1.15, 1.24, and 1.41) in the adjusted prevalence ratios of severe sleep disorders linked to a higher number of attack or threat against correctional officers over the last 12 months increased. Further, higher prevalences of severe sleep disorders were strongly associated with the number of stressful activities at work. Work is considered a strong societal determinant of sleep quality^26^. These situations of tension and violence can act as stressors in the correctional officers’ daily working lives, and worsen their sleep quality. In a qualitative research about work conditions and their impact on health, correctional officers reported that sickness was due to moments of tension experienced in the prison, causing fear, insecurity, and dissatisfaction^27^.

Brazilian prisons face steady growth in the number of criminal factions and difficulties in controlling them inside prisons^28^. Correctional officers’ close contact with members of criminal factions affects their health, especially their mental health, and contributes to poor sleep quality. Bivariate analysis revealed a 32% higher prevalence among correctional officers who had been attacked or threatened by faction members. Attacks and threats perpetrated by prisoners linked to criminal factions have certainly contributed to the variable “number of attack or threat events against the correctional officer over the last 12 months” which, for its part, was strongly associated with severe sleep disorders.

Poor working conditions and specific features of their occupation may have contributed to the strong association (RP=2.43) between common mental disorders and severe sleep disorders found in this study. Correctional officers present a high prevalence of mental health problems^1^. A study carried out in 1999, with a sample of 311 correctional officers from the State Penitentiary Complex of Salvador, using SRQ-20 with the 7/8 cutoff point, reported a 30.7% prevalence of common mental disorders, strongly associated to multiple adverse working conditions^29^. The SRQ-20 cutoff >7 used in the 1999 survey for both sexes has certainly underestimated the prevalence of common mental disorders. Furthermore, the proportion of female correctional officers in the institution has increased in the period 1999-2018 from 8.0% to 16.6%.

The adjusted prevalence of severe sleep disorders was 37% higher among officers declaring their skin color to be non-White compared to the White. Adults from race/ethnicity White presented better sleep quality^26,30^ than Afro-American and Latinos, and higher rates of long and short sleep^31,32^ than their Afro-American counterparts.

The adjusted prevalence of severe sleep disorders among correctional officers who worked shifts was only 15% higher than among those who did not. In this penitentiary complex, work schedules are organized in 24-hour shifts followed by a 72-hour rest. However, double shifts are frequent, mainly due to shift changes or substitutions between correctional officers. Shift work can be harmful to the sleep quality of correctional officers, particularly when associated with long working hours^32^.

Selection bias is one notable study limitation, since only 83.7% of the eligible population was investigated. However, the 27 correctional officers excluded because of incomplete data presented characteristics similar to those found among the 374 individuals effectively investigated. Another limitation of this study was the lack of an obesity measure^33^, because of its possible association with severe sleep disorders. Due to its cross sectional design, this study has only limited capacity to establish causality. Except for skin color, all other independent variables that were found associated with severe sleep disorders could have had a reverse causality relationship with this dependent variable. Furthermore, sleep is hard to measure, because of its subjective nature^34^. Studies use several instruments to assess sleep and only a few of them have used the *mini sleep questionnaire*, limiting the comparison of results. The small number of studies about sleep quality among correctional officers also prevent comparison. Future studies, using more acurate methods to assess sleep, such as actigraphy and polysomnography, can throw new light on this issue.

In conclusion, this study found a high prevalence of severe sleep disorders among correctional officers, associated with stress and violence at work, common mental disorders, and skin color. These factors must be taken into account in future actions aiming to provide health care to these workers. Hopefully, stress and violence at work are possible to be modified. In order to improve correctional officers sleep quality, the prison complex management and the occupational health service personnel should consider improving the workplace environment and physical and psychosocial working conditions.
